# Editorial: Antimicrobial resistance in zoonotic bacteria in developing countries: the role of food animal production in public health, volume II

**DOI:** 10.3389/fvets.2023.1295971

**Published:** 2023-10-23

**Authors:** Josefina León-Félix, Rafael Vignoli

**Affiliations:** ^1^Laboratorio de Biología Molecular y Genómica Funcional, Centro de Investigación en Alimentación y Desarrollo (CIAD), Culiacán, Mexico; ^2^Departamento de Bacteriología y Virología, Instituto de Higiene, Facultad de Medicina, Universidad de la República, Montevideo, Uruguay

**Keywords:** antimicrobial resistance, zoonotic bacteria, food animal, public health, developing countries

Antimicrobial resistance is one of the main health challenges of the current decade according to the WHO (1). It is estimated that globally, more than 70% of antibiotics are used in the production of food of animal origin (2).

According to the 6th Annual Report on Antimicrobial Agents Intended for Use in Animals of the World Organization for Animal Health (WOAH), the main families of antimicrobials used in food production are tetracyclines, penicillins, and macrolides (3). However, the detection of microorganisms resistant to antibiotics critical to humans in food-producing animals is a growing problem (4).

In this Research Topic, contributions can be separated into two groups, on the one hand the studies regarding the antimicrobial resistance detected in potentially zoonotic microorganisms isolated from food-producing animals and, on the other hand, a set of research related to the analyses, practices and attitudes in the management of antimicrobial resistance in the animal sector.

Thus within the first group, Gugsa et al. describe the microbiological characteristics of *Escherichia coli* O157:H7 isolated from foods of bovine origin in Ethiopia. They study a total of 284 samples [raw milk (*n* = 145), yogurt (*n* = 48), and meat (*n* = 91)] from which *E. coli* was detected in 24.6% of samples and Shiga toxin-producing *E. coli* O157:H7 was identified in 14.3%, resistance to penicillins (90%−95%), macrolides (54%) and tetracyclines (40%−50%).

Retamal et al. describe the presence of virulence genes and antimicrobial resistance patterns in 46 isolates of *Salmonella enterica* isolated from 500 fecal samples from pigs and 57 isolates form 300 fecal samples of chickens in central Chile. The most frequently isolated serovars were *Salmonella* Typhimurium, *Salmonella* Infantis, *Salmonella* Enteritidis, and *Salmonella* Derby. The isolates obtained from chickens were more multiresistant (82.5%) than those isolated from pigs (8.7%), with resistance to quinolones, first generation cephalosporins and sulfonamides prominent in the former, and resistance to tetracycline and sulfonamides in the latter. Almost 30% of the isolates obtained from chickens were resistant to oxyminocephalosporins, whereas such resistance was not observed in isolates from pigs.

Finally, Casaux et al. compare antibiotic susceptibility results determined by phenotypic methods against those predicted by whole genome sequencing of 75 *S. enterica* isolates from dairy cattle and dairy farm environments. *S*. Typhimurium, *Salmonella* Newport, *Salmonella* Anatum, and *Salmonella* Dublin was most frequently serovars. Phenotypic resistance to streptomycin was the most frequently detected (85.3%) followed by a non-susceptibility to ciprofloxacin (77.3%) and tetracycline (67%).

The *tetA, aph (3”)-Ib, aph (7)-Id* array were detected in 21 isolates associated with an 85 kb IncFII plasmid and *tetA, aph (3”)-Ib, aph (7)-Id, sul2* were detected in 19 isolates on an 8.5 kb cryptic plasmid. The accuracy of predicting antimicrobial resistance phenotypes based on AMR genotypes was 83.7%.

In the second group of articles, Caipo et al., developed a methodology for a qualitative evaluation of risk factors with the objective of providing a qualitative and systematic assessment of AMR risk. The results of the information analysis made it possible to develop a roadmap guiding and prioritizing sectoral needs and actions for the containment of AMR under an intersectoral, multidisciplinary and collaborative approach, and in accordance with the priorities and resources of the countries, they concluded that the methodology is useful to define and prioritize risk factors and opportunities to reduce AMR in the animal production sector. On the other hand, Ting et al., conducted a study with the purpose of exploring knowledge about the attitudes and practices regarding antibiotic use and antibiotic resistance of government animal health workers in Timor-Leste. The study identified poor knowledge about antibiotics, only 8.0% were able to correctly answer questions about how antibiotics work, knowledge about antibiotic resistance was poor as only 29.0% had heard of antibiotic resistance and were able to accurately identify what made antibiotics less effective, knowledge about antibiotics and their resistance was associated with being a veterinary technician and having a university education, attitudinal assessments were positively influenced by knowledge about antibiotics and antibiotic resistance, regarding the use of antibiotics, the empirical use of antibiotics in sick animals predominated and the use of antibiotics to promote growth was uncommon. This study concluded that specific treatment guidelines for Timor-Leste improve veterinary diagnostic support, appropriately manage antibiotics and develop training programs are necessary to fill knowledge gaps and poor practices found in this study. For their part, Vijay et al., developed a study to monitor antimicrobial use (AMU) in adult cattle in India, using the “container method” which is based on manual collection of empty medicine containers. The authors concluded that the method used offers a more accessible alternative to AMU monitoring that allows recording the actual consumption of antimicrobials and provides an overview of the qualitative and quantitative estimation of AMU among adult Indian cattle. Finally, Tufa et al., developed a study to understand the attitude and behavior of livestock producers regarding antimicrobial use (AMU) in Ethiopia, with the objective of understanding the mentality of farmers to improve the management of antimicrobials in the livestock sector in Ethiopia. The results of the study conclude that low level of awareness and undesirable attitudes toward AMU and AMR could potentially affect farmers' behavior toward judicious AMU, which would require awareness creation efforts on livestock disease management practices.

## Author contributions

JL-F: Writing—original draft, Writing—review and editing. RV: Writing—original draft, Writing—review and editing.
